# Plasma hemoglobin concentration among pregnant and non-pregnant women in Mwanza: are we using correct reference values to diagnose anemia in pregnancy?

**DOI:** 10.11604/pamj.2018.30.93.11954

**Published:** 2018-05-31

**Authors:** Haruna Dika, Elizabeth Masawe, Shabani Iddi, Richard Rumanyika

**Affiliations:** 1 Department of Physiology, Weill Bugando School of Medicine, Catholic University of Health and Allied Sciences, Mwanza, Tanzania; 2 Department of Obstetrics and Gynaecology, Bugando Medical Center, Mwanza, Tanzania; 3 Archibishop Antony Mayalla School of Nursing, Catholic University of Health and Allied Sciences, Mwanza, Tanzania; 4 Department of Obstetrics and Gynaecology, Weill Bugando School of Medicine, Catholic University of Health and Allied Sciences, Mwanza, Tanzania

**Keywords:** Plasma haemoglobin concentration, healthy women, African Tanzanians

## Abstract

**Introduction:**

The definition of anemia has attracted considerable interest because several studies have demonstrated that hematologic profile vary with ethnicity in addition to age, sex and altitude. This has led scholars to recommend the use of population specific hematologic reference values in diagnosing blood disorders. However, there is limited information about Tanzanians population specific hemoglobin (Hb) levels which can be used to set cut-off points to define anemia. This study aimed to determine plasma Hb concentrations among healthy Tanzanian women.

**Methods:**

This cross sectional study was done in Mwanza. Sociodemographic data were collected using questionnaires and plasma Hb concentrations were measured by calorimetric method. Data were analyzed using SPSS.

**Results:**

A total of 215 (162 pregnant, 53 non-pregnant) women with a mean age of 28.2 ± 6.54 years participated in the study. The mean plasma Hb concentrations were 12.0 ± 1.43 mg/dl and 11.9 ± 1.15 mg/dl for pregnant and non-pregnant women respectively. The Hb levels did not significantly vary between pregnant and non-pregnant women. Using WHO reference values, 45.3% non-pregnant and 26.5% pregnant women were found to be anemic while using the population specific reference, only 1.9% of pregnant and none of non-pregnant women would be classified as anemic.

**Conclusion:**

Most Tanzanian women who are diagnosed to have anemia during pregnancy, often had developed lower Hb before pregnancy and operational thresholds for diagnosis of anemia observed in this study are lower than WHO recommended references values. We recommend a large scale study to determine hematological profile of Tanzanian.

## Introduction

In many African countries including Tanzania the definition of anemia is based on reference values of hemoglobin level set by World Health Organization (WHO) nearly 50 years ago using data collected from developed countries [1, 2]. While the same hematologic reference ranges are routinely applied to patients with different ancestral origins, for several decades now, it is known that there are differences, particularly between “normal” values obtained from different ethnic populations [3-13]. Studies in the United States of American clearly demonstrated that whites have significantly greater mean hemoglobin concentrations than blacks of the same age and sex and living in the same environment [6, 9, 11, 12]. Other studies which showed that ethnicity is an important factor in interpreting hematologic profile of individuals are those which demonstrated that Han people who migrated to the Qinghai-Tibetan Plateau have hemoglobin concentrations higher than native Tibetans [3-5, 7-10, 13]. These Qinghai-Tibetan Plateau study findings suggest that there is an ethnic difference in the erythropoietic response to high altitudes [7, 8, 10, 13]. Medical decisions made by physicians regarding investigation of anemia, usually depend on whether the patient's hemoglobin level falls within the accepted reference range. The differences in Hb levels between populations with different ancestral origin derived the scholars to suggest a consideration of using separate hematologic reference values for each population [9, 11, 12], because choosing unrelated "normal ranges" may lead to a false diagnosis and unnecessary additional investigation and treatment costs [12].

When these ranges are derived from one population and then applied to another, unnecessary investigations of seemingly aberrant laboratory results may be the consequence. For instance in a study done by Beutler and West, about 20% of the healthy African-American women were judged anemic based on general population's reference range [6] but if population specific reference range was used, this would have not been the case. Furthermore, the WHO estimates, show that, up to 56% of all women particularly pregnant ones living in developing countries are anemic [14]. However it is not known whether pregnant women in developing countries like Tanzania who are largely diagnosed as anemic, develop anemia during pregnancy or they are usually anemic even before getting pregnant. The use of inappropriate evaluative hemoglobin criteria during pregnancy can result in misinterpretation of the relation between anemia and associated birth outcomes. Despite of scholars' suggestions that, the correct interpretation of Hb or haematocrit values requires the consideration of ethnicity and other local factors in selecting appropriate cut-off values, there is limited information regarding the normal haematologic profile which was locally derived in African countries. For example, the current reference ranges for normal Hb levels by age, sex and altitude which are used in Tanzania were derived from American and European populations [1, 2]. These reference values may not be normal for African and particularly Tanzanian population. In addition to age and sex, Tanzanian physicians take into account environmental factors particularly altitude when interpreting Hb level of individuals [2, 15] but ethnicity is not considered at all. High prevalence of anemia during pregnancy reported for African countries could also be a result of exaggeration due to the use of inappropriate plasma Hb concentrations reference values. Therefore, this study determined plasma Hb concentration among healthy pregnant and non-pregnant women in Mwanza city. Results from this study give an insight about whether the relevance of reference range used in our setting to diagnose anemia in pregnancy.

## Methods

**Study design**: This was a cross sectional descriptive study.

**Study setting**: The study was conducted at Makongoro Clinic, a primary health care facility located in Nyamagana District within Mwanza City, Tanzania. The city is located on the southern shores of Lake Victoria in the northwest part of Tanzania. The clinic serves mainly two districts, namely Nyamagana and Ilemela.

**Participants**: The study involved pregnant women attending antenatal clinic (ANC) for the first time and non-pregnant female relatives who accompanied them.

**Inclusion criteria**: The study included only subjectively healthy Tanzanian women of child bearing age (15 years to 45 years).

**Exclusion criteria**: Non-African (non-black) Tanzanians were not enrolled in this study. Women who reported to be known or who were clinically identified to have acute or chronic illness were excluded from the study. Women with symptoms or signs of anemia and those on iron or folic acid supplements were also excluded from study.

**Sample size**: A minimum of 154 pregnant women attending Makongoro ANC for the first time were required. The minimum sample size was determined using Taro Yamane formula [16] with 95% confidence level. The calculation formula of Taro Yamane is presented as follows:

n=N/1+N(e)^2^

where: n = sample size required, N = number of people in the population, in this case is new pregnant women attending Makongoro Clinic who were estimated to be 250 for the period of three months, e = allowable error (%)-0.05. Substituting in the formula: n = 250/1+250(0.05)^2^ = 153.8. All non-pregnant women relatives who met inclusion criteria and voluntarily agreed to participate in the study were recruited.

**Sampling procedures**: A consecutive sampling procedure was used to include subjects that met the inclusion criteria.

**Data collection**: Data was collected from November 2015 to January 2016. Structured questionnaire was used to collect socio demographic data. Five ml of fresh venous blood was drawn from cubital vein of each participant using sterile vacutainer needle. Blood was stored at room temperature before estimation of plasma Hb concentration which was done within 24 hours. Drabkin's cyanmethemoglobin method was used to measure plasma Hb concentration using Biochrom WPA CO7000 Medical Colorimeter (Biochrom Ltd). Drabkin's cyanmethemoglobin is the calorimetric method whose underlying principle is the formation of cyanmethemoglobin, a stable color pigment which is read photometrically [17, 18]. Measurement of plasma Hb was done following procedures as earlier described by Dallman and Bothwell [19]. In brief, the Drabkin's solution containing potassium ferricyanide, potassium cyanide and sodium bicarbonate was prepared and 5 mls of it was mixed with 20 µl of blood. When the Drabkin's solution is mixed with blood, the potassium ferricyanide oxidizes Hb iron to form methemoglobin. The potassium cyanide then combines with methemoglobin to form cyanmethemoglobin, which is read photometrically at a wave length of 540nm. Tubes were allowed to stand for 10 minutes before taking the readings. Hb values were obtained comparing the read absorbance (optical density) to the corresponding values on a hemoglobin curve prepared using Hb standard.

**Data analysis**: Statistical analyses were done using Statistical Package for the Social Sciences (SPSS) software version 17. Categorical data were cleaned, edited, coded and entered into Microsoft Excel together with continuous data. Data were then exported to SPSS for analysis. Plasma Hb concentrations are expressed in ranges and mean ± standard deviation (SD) and summarized in tables. Comparison of mean Hb concentrations by various independent variables was done by repeated measures analysis of variance (ANOVA). When significant ANOVAs were obtained, post-hoc analysis was performed using Tukey's Multiple Comparison Testing. Statistical significance levels were fixed at two-tailed p-value of 0.05.

**Ethical considerations**: Ethical clearance was sought and provided by the joint Catholic University of Health and Allied Sciences and Bugando Medical Centre Research Ethics and Review Committee. Permission to collect data was granted by City Medical Officer and Makongoro Clinic administration. Participants voluntarily participated in the study and they (or their guardians) signed written informed consents.

## Results

**Characteristics of study participants**: A total of 215 women, with the mean age of 28.2 ± 6.54 years (range 16-45 years) participated in this study. Among them, 162 (75.3%) were pregnant women attending ANC and 53 (24.7%) were non-pregnant female relatives. 179 (83.3%) of the participants, were from urban areas while 36 (16.7%) were from suburb areas. Subjects belonged to varying tribes with majority of them being Sukuma followed by Haya, Kurya, Ha (also called Abaha) and Chagga.

**Plasma haemoglobin concentration**: The plasma Hb concentration ranged from 8.0-16.5 mg/dl with a mean of 12.0 ± 1.43 mg/dl. There was no significant (p = 0.931) difference in Hb levels between pregnant women and non- pregnant women (Table 1). There was no significant difference in Hb levels between participants from urban and suburb areas. Mean plasma Hb concentration was 11.9 ± 1.37 mg/dl and 12.3 ± 1.66 mg/dl for participants from urban and suburb areas respectively (p = 0.090). The mean plasma Hb concentrations did not significantly (p = 0.324) vary with age of the women (Table 2). Furthermore, there was no significant difference (p = 0.430) in Hb level between women of varying tribes (Table 2).

**Table 1:** Plasma hemoglobin concentration of non-pregnant and pregnant women

State	Mean Hb Concentration (mg/dl)	Acceptable Lower Limit, mg/dl (Mean -2SD)
**Non-pregnant (n=53)**	11.9 ± 1.15	9.6
**Pregnant**		
1^st^ trimester (n=39)	12.1 ± 1.22	
2^nd^ trimester (n=78)	12.0 ± 1.44	
3^rd^ trimester (n=45)	11.9 ± 1.85	
**Pregnant-All**(n=162)	12.0 ± 1.43	9.0

1^st^ trimester = ≤ 12 weeks pregnancy, 2^nd^ trimester = 13 – 27 weeks pregnancy, 3^rd^ trimester = ≥ 28 weeks pregnancy. F(0.147), df 3, p = 0.931

**Table 2:** Plasma hemoglobin level by age and tribes

Group of subjects	Hb concentration (mg/dl)
***Age group***	
<20 (n=22)	11.5 ± 1.62
20-24 (n = 49)	11.9 ± 1.69
25-29 (n = 62)	12.1 ± 1.33
30-34 (n = 53)	12.2 ± 1.26
35-39 (n = 20)	12.1 ± 1.25
40+ (n = 9)	11.4 ± 1.16
Total (n = 215)	12.0 ± 1.43
***Tribes***	
Sukuma (n = 79)	11.9 ± 1.52
Haya (n = 41)	11.8 ± 1.30
Kurya (n = 26)	12.3 ± 1.42
Chagga (n = 12)	11.8 ± 1.46
Ha (n = 13)	12.7 ± 1.13
Hehe (n = 6)	11.8 ± 1.79
Others (n = 38)	12.1 ± 1.38
Total (n = 215)	12.0 ± 1.43

F(1.171), df 5, p = 0.324 (across age groups); F(0.994), df 6, p = 0.430 (across tribes)

**Anemic status**: Based on WHO reference values, 43 out of 162 (26.5%) pregnant and 24/53(45.3%) non-pregnant women who participated in this study had mild to moderate anemia (Table 3). However, using the acceptable lower limits derived from the current study (Mean-2SD) only 3 (1.9%) of pregnant women would be classified as anemic (Hb < 9.0 gm/dl) and none of non-pregnant women would be classified as anemic (Hb < 9.6 gm/dl).

**Table 3:** Classification of study participants on their anemic status

Subjects	Anemic State	Reference Hb (gm/dl)	Frequency	Percentage
**Pregnant**	Non-anemic	≥ 11	119	73.5
	Mild Anemia	10 – 10.9	32	19.7
	Moderate Anemia	7 – 9.9	11	6.8
	Severe Anemia	< 7	0	0.0
	Total		162	100
**Non-Pregnant**	Non-anemic	≥ 12	29	54.7
	Mild Anemia	11 – 11.9	9	17.0
	Moderate Anemia	8 – 10.9	15	28.3
	Severe Anemia	< 8	0	0.0
	Total		53	100

None of the participant had severe anemia

## Discussion

This study has determined plasma level of hemoglobin (Hb) among pregnant (12.0 ± 1.52 mg/dl) and non-pregnant (11.9 ± 1.15 mg/dl) women of Mwanza city which may be used as reference values for Tanzanian women. The Hb levels obtained did not show significant variation between pregnant and non- pregnant women. Likewise, they did not significantly vary by age and between women of varying tribes. As per WHO reference values, 26.5% of pregnant and 45.3% of non-pregnant women who participated in this study were found to have mild to moderate anemia. Lack of variation in Hb levels among women of different tribes observed in the present study is in contrary with previous knowledge that hematologic profile including Hb concentration vary among subjects of different ethnic groups [5, 6, 8-12]. For instance, in the United States, individuals of African-American have been found to have lower average hemoglobin level, hematocrit and mean corpuscular volume compared to whites, the difference which was not related to environmental factors [6]. Small sample size in the present study could be a reason for lack of statistical significance effect. Intermarriage among Tanzanians of different tribes is also a likely explanation for observed lack of difference in mean Hb concentrations between tribes. So, what we considered as tribal groups are not purely single tribes but a mixture of tribes.

The fact that, the study participants were living in almost similar geographical environment namely altitude which is the main determinant of erythropoiesis [2, 3], could also account to lack of variation in Hb by tribal differences in the current study. Subjects of various tribal groups could have their erythropoietic activity changed to adapt to altitude. Contrary to the previous knowledge that plasma hemoglobin concentration tends to decrease during pregnancy [1, 2, 20, 21], the current study has found lack of significant difference in mean Hb concentrations between pregnant and non-pregnant women. Furthermore, despite of excluding women who were on iron and/or folic acid supplements, relatively many non-pregnant women were found to be anemic compared to pregnant ones. Pregnant women are likely to eat more nutritious foods and they frequently use a number of traditional herbs than non-pregnant ones. Therefore good nutrition and the use of local herbs which may have haematenic effects among pregnant women could explain lack of difference in mean Hb levels and small prevalence of anemia among pregnant women compared to non-pregnant ones. Additionally, the proportion of women who were diagnosed to be anemic in the present study is lower than WHO estimates. WHO estimated that, up to 56% of all women particularly pregnant ones living in developing countries are anemic [14]. The lower prevalence of anemia in the current study compared to WHO estimates is due to exclusion of obviously sick women such as those who had symptoms and/or signs of anemia.

## Conclusion

Based on our findings, we can conclude that Tanzanians of various tribes have nearly similar mean hematological profiles. Our findings suggest that most pregnant women in Tanzania who are diagnosed to have anemia usually had anemia before pregnancy, which is likely to be nutritional anemia. We also conclude that, fewer women would be classified as anemic if population specific acceptable reference values are used compared to when WHO recommended reference values are used. We recommend a large scale study to determine hematological profile of Tanzanians. We also recommend nutritional education to be provided to the citizens particularly women of reproductive age so as to avoid nutritional anemia.

### What is known about this topic

- Lower limit of plasma Hb concentrations used for diagnosis of anaemia are 11 gm/dl and 12 gm/dl for pregnant and non-pregnant women respectively;
- Plasma Hb levels vary with ethnicity whereby levels are higher among Caucasians than Africans;
- About 56% of women in developing countries are anemic and mostly during pregnant.

### What this study adds

- Operational thresholds of plasma Hb levels for diagnosis of anemia (mean-2SD) among Tanzanian women are lower than WHO recommended references values which were set about 50 years ago;
- Most Tanzanian women who are diagnosed to have anemia during pregnancy are likely to have developed lower Hb levels even before pregnancy.
